# Editorial: Translational research in medical robotics—challenges and opportunities

**DOI:** 10.3389/frobt.2023.1270823

**Published:** 2023-10-04

**Authors:** Giulio Dagnino, Dennis Kundrat, Pedro Moreira, Helge Arne Wurdemann, Momen Abayazid

**Affiliations:** ^1^ Robotics and Mechatronics, University of Twente, Enschede, Netherlands; ^2^ Brigham and Women’s Hospital, Harvard Medical School, Boston, MA, United States; ^3^ Department of Mechanical Engineering, University College London, London, United Kingdom

**Keywords:** medical robotics, translational research, user studies, clinical studies, results reproducibility

In the last few decades, emerging medical technologies and the growing number of commercial robotic platforms have supported diagnosis and treatment of both acute and chronic diseases of the human body, improving the clinical outcome, reducing trauma, shortening the patient recovery time, and increasing postoperative survival rates (Troccaz et al., 2019). Medical robots–including surgical robots, rehabilitation and assistive robots, and hospital automation robots–with improved safety, efficacy and reduced costs, robotic platforms will soon approach a tipping point, moving beyond early adopters to become part of the mainstream clinical practice, defining the future of smart hospitals and home-based patient care. Surgical robots promise to enhance minimally invasive surgery with precise instrument control, intuitive hand-eye coordination, and superior dexterity within tight spaces (Dupont et al., 2021). Rehabilitation robotics facilitates robot-assisted therapy and automated recovery training (Xue et al., 2021). Assistive robots aid individuals with physical limitations, either enhancing or compensating for functions, promoting independence, and lessening the burden on caregivers (Trainum et al., 2023). Additionally, robotic systems can automate hospital operations, spanning service robots aiding clinicians to robots in labs for high-throughput testing (Kwon et al., 2022). These technologies aim to revolutionize healthcare, offering improved patient care and operational efficiency.

The commercial success of medical robotic platforms, is the outcome of continuous efforts in translational research on novel medical devices. This pathway usually starts with an initial idea related to a clinical need or challenge and targets its long-term translation into a clinically approved device. Selected milestones along this path predominantly aim at increased technical maturity of existing laboratory demonstrators or proofing feasibility in relevant preclinical/clinical environments involving end users. These steps are essential building blocks for prospective clinical clearance and approval processes.

Although properties of translational roadmaps are comparable among projects, individual contributions to requirements, timelines, resources, costs and procedures may differ significantly. Yet, major challenges in translational research not only arise from securing long-term project funding, but also from holistic consideration of complex and dynamic ethical and regulatory aspects (Yang et al., 2018).

This Research Topic provides a useful overview of some key aspects involved in the translational journey of medical robots, including human-robot interaction and robot autonomy, benchtop trials, clinical testing, and validation challenges. The goal is to provide a roadmap for successful clinical translation, addressing clinical opportunities, technical requirements and regulatory challenges for translating robots to practical clinical use.

Translating medical robots into practice requires the systems to be easily integrated in the clinical workflow and to cooperate with the clinical teams. Typically, robotic platforms that offer little or no autonomy and are controlled remotely by the clinician are more readily accepted. However, for certain intricate or monotonous tasks, it may be possible to assign complete responsibility to the robot. McDonald-Bowyer et al. developed sensing and control strategies to perform autonomous intra-operative ultrasound scans on kidneys using the da Vinci system during robot-assisted partial nephrectomy. The hypothesis the authors present is that automating this challenging sub-task may reduce the cognitive load for the surgeon improving patient outcomes. The study demonstrates the approach feasibility through benchtop experiments conducted on an artificial kidney phantom.

Benchtop trials serve as a crucial initial step to evaluate a newly developed technology; however, clinical trials are indispensable in order to validate and implement the technology in a real-world clinical setting. Barria et al. have developed the RobHand a robotic neuromotor rehabilitation exoskeleton that assists in performing flexion and extension movements of the fingers. The authors have tested the RobHand on four chronic stroke patients, to evaluate the safety, rehabilitation capabilities and usability of the robot. While the trials demonstrated the device safety, no statistically significant improvements were found in manual motor function due to the low number of patients recruited. In future studies, the scope will be expanded to include a larger sample size. Additionally, there is a possibility of involving patients with various nervous and musculoskeletal conditions, such as spinal cord injury, peripheral neural injuries, as well as individuals undergoing rehabilitation for traumatic and post-operative musculoskeletal hand injuries.

One of the significant challenges in the translation of medical robot prototypes is ensuring the reproducibility and benchmarking of testing results. This step is crucial to obtain the required regulatory approval for market introduction. Faragasso and Bonsignorio highlight the need for developing new tools and putting in place a community effort to allow the transition to more reproducible research and hence faster progress in research. The authors have selected 10 relevant published manuscripts on surgical robotics to analyze their clinical applicability and underline the problems related to reproducibility of the reported experiments. The study showed that the selected experimental papers are very often missing features that would allow independent researchers to reproduce the described work and compare the results, showing a need to overcome this limitation to avoid flaws and inconsistencies.

This Research Topic provided an insight into selected technical and clinical challenges that need to be resolved, striving to further consolidate the collaboration between the clinical, engineering and regulatory communities.
